# Oxidative Modifications in Advanced Atherosclerotic Plaques: A Focus on *In Situ* Protein Sulfhydryl Group Oxidation

**DOI:** 10.1155/2020/6169825

**Published:** 2020-01-07

**Authors:** Antonio Junior Lepedda, Marilena Formato

**Affiliations:** Dipartimento di Scienze Biomediche, University of Sassari, Sassari, Italy

## Abstract

Although oxidative stress has been long associated with the genesis and progression of the atherosclerotic plaque, scanty data on its *in situ* effects on protein sulfhydryl group modifications are available. Within the arterial wall, protein sulfhydryls and low-molecular-weight (LMW) thiols are involved in the cell regulation of both Reactive Oxygen Species (ROS) and Reactive Nitrogen Species (RNS) levels and are a target for several posttranslational oxidative modifications that take place inside the atherosclerotic plaque, probably contributing to both atherogenesis and atherosclerotic plaque progression towards complicated lesions. Advanced carotid plaques are characterized by very high intraplaque GSH levels, due to cell lysis during apoptotic and/or necrotic events, probably responsible for the altered equilibrium among protein sulfhydryls and LMW thiols. Some lines of evidence show that the prooxidant environment present in atherosclerotic tissue could modify filtered proteins also by protein-SH group oxidation, and demonstrate that particularly albumin, once filtered, represents a harmful source of homocysteine and cysteinylglycine inside the plaque. The oxidative modification of protein sulfhydryls, with particular emphasis to protein thiolation by LMW thiols and its association with atherosclerosis, is the main topic of this review.

## 1. Introduction

Cardiovascular diseases are the leading cause of death and illness in developed countries, being atherosclerosis a major contributor [1]. Cardiovascular risk factors such as hypertension, diabetes, and hyperlipidaemia play a key role in the onset and progression of atherosclerosis [2]. Atherosclerosis, a chronic inflammatory condition, develops and evolves in a site-specific and patient-specific manner, with a great heterogeneity in growth rate and pathologic features [3]. Atherosclerotic plaques are commonly characterized by accumulation of lipids and fibrous elements in the intimal layer of medium size and large arteries. Erosion of the atherosclerotic plaques could cause plaque ulceration leading to acute thrombosis and artery occlusion, driving major adverse clinical events. Indeed, plaque disruption is a common precipitating factor in the pathogenesis of both acute coronary occlusion and peripheral artery thrombosis. The mechanisms underlying plaque formation and progression towards advanced lesions potentially prone to rupture are not yet completely understood. Despite current systemic application of therapies, such as statins and antiplatelet agents for prevention of both accelerated plaque growth and thrombotic consequences of its rupture, most of major adverse cardiovascular events cannot be averted [3].

Understanding the mechanisms that promote thin fibrous cap formation and stabilization, as opposed to lysis and disruption, would help to effectively counteract the release of prothrombogenic elements and prevent acute thrombotic occlusion. It is generally held that plaque instability is caused by a substantial increase in inflammatory and proteolytic activity [4]. Furthermore, some lines of evidence suggest that unstable plaques are also characterized by pronounced oxidative environment [5]. *In situ* oxidative events may determine lipid/protein metabolic fate, bioactivity, and antigenic properties.

This review will focus on oxidative modifications of protein sulfhydryl groups, with particular emphasis to protein thiolation by low-molecular-weight thiols (LMW thiols), and their association with atherosclerosis.

## 2. Oxidative Stress and Atherosclerosis

A great deal of research has shown a contributory role of oxidative modifications of apolipoprotein B-100-containing lipoproteins (LDL and Lp(a)), within the arterial wall, in the early events of atherogenesis [1, 6, 7]. Oxidized LDL is readily internalized by macrophages through the so-called “scavenger receptor” pathway. These early modifications could initiate and/or contribute to atherogenesis, mainly when an imbalance between oxidant and antioxidant agents takes place [7]. Although several studies report that atherosclerotic plaques contain high concentrations of some amino acid oxidation products, caused mainly by carbonylation, ROS and RNS oxidation, or thiolation [8–10], limited information is available regarding the relationship between the accumulation of *in situ* oxidized proteins and atherosclerosis severity [11]. Furthermore, different oxidation-specific epitopes can be detected in blood and may reflect atherosclerosis manifestations [12]. At present, the mechanisms underlying the formation of these by-products and their relevance for disease progression are not completely understood and deserve further investigation.

ROS and RNS are highly reactive electrophiles that oxidize nucleophilic functional groups of proteins, polysaccharides, and nucleic acids such as -OH, -NH_2_ and -SH, leading to cell damage and death, if not properly repaired. Due to its chemistry, -SH is more reactive than -OH and -NH_2_ and, consequently, more prone to oxidation or conjugation [13, 14].

Within the arterial wall, several enzymes as well as nonenzymatic antioxidants take part in counteracting these oxidative modifications [7]. Among them, protein sulfhydryl groups (protein-SH (PSH) groups) and low-molecular-weight thiols (LMW thiols) are involved in the cell regulation of both ROS and RNS levels. Due to its high reducing power and its relatively high concentration, glutathione (GSH) represents the best intracellular reducing agent. In this respect, it has been reported that human atherosclerotic plaques display lack of GSH-peroxidase (Px) activity and a deficient glutathione redox cycle status that may significantly weaken its antioxidant potential [15, 16], so corroborating the hypothesis that the prooxidant environment within the vascular wall might be involved in atherogenesis and complicated lesion formation. Further evidences come from differential proteomic analysis on advanced unstable and stable carotid plaques, where a reduced expression of superoxide dismutase 3 (major defence against the superoxide anion radical in the vascular extracellular matrix) and glutathione S-transferase (vessel protection against reactive species such as *α*,*β*-unsaturated carbonyls and 4-hydroxy-2-nonenal), in the former, suggests an even more impaired antioxidant/prooxidant balance in those plaques more prone to rupture [17].

## 3. Protein Sulfhydryls Oxidation and Atherosclerosis

**Figure 1:**
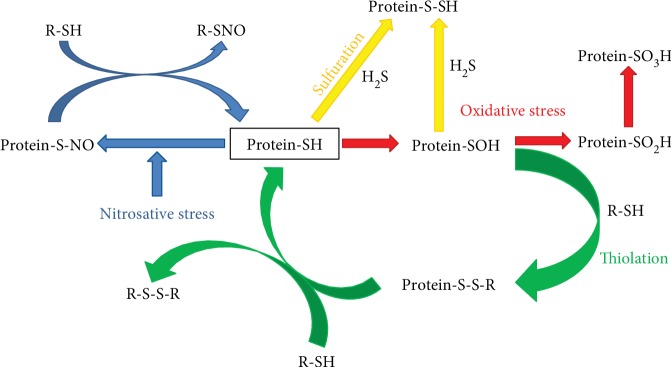
Overview of the wide variety of biochemical modifications that reduced protein sulfhydryl groups may potentially undergo. R-SH or LMW thiols: *γ*Glu-Cys-Gly (*γ*glutamyl-cysteinil-glycine, glutathione), Cys (cysteine), HCy (homocysteine), Cys-Gly (cysteine-glycine), and Glu-Cys (glutamyl-cysteine).

The reactivity of LMW thiols, namely cysteine-glycine (Cys-Gly), homocysteine (HCy), cysteine (Cys), glutathione (GSH), and glutamylcysteine (Glu-Cys), due to their proton lability (pKa), is strictly dependent on their structure, whereas reactivity of protein-SH is affected also by the exposure to the milieu [18]. Some proteins, such as albumin and haemoglobin, have reactive cysteine residues susceptible to some reversible (thiolation, nitrosylation, and sulfenylation) or irreversible (sulfinilation and sulfonation) oxidative modifications (Figure 1). In the last years, it is becoming increasingly clear that also protein S-sulfuration [19] represents an important mechanism of regulation of protein activity mediated by hydrogen sulfide (H_2_S) (Figure 1). In this respect, it has been reported that these modifications occur on enzymes, receptors, transcription factors, and ion channels and represent key regulatory events in maintaining the physiological function of proteins in the cardiovascular system [20].

Among the abovementioned reversible reactions, protein S-thiolation by LMW thiols is the most biologically stable and important one [21]. The formation of S-thiolated proteins represents an antioxidant defence mechanism against such reactive molecules, and it has been suggested as a possible redox regulation mechanism of protein function [22, 23] and cell signalling [24, 25]. Indeed, the reversible covalent modification of some protein cysteine residues may be transitory and have critical modulatory effects as suggested for the activation of the latent elastolytic metalloproteinase-2 (pro-MMP-2) by homocysteinylation and S-glutathionylation of the propeptide via the so-called “cysteine switch” mechanism [26–29]. The activation of matrix metalloproteinases (MMPs) by LMW thiol adduction may have a key role in the extracellular matrix degradation and plaque rupture. Furthermore, reversible oxidative modifications, including sulfenylation, S-nitrosylation, and S-thiolation are thought to act as redox sensors in cell signalling pathways [30–32].

S-Glutathionylation is emerging as having a causative role in cardiovascular diseases by regulating numerous physiological processes involved in cardiovascular homeostasis, including myocyte contraction, oxidative phosphorylation, protein synthesis, vasodilation, glycolytic metabolism, and response to insulin [33]. With respect to atherogenesis, there are several in vitro evidences that GSH protects macrophages from OxLDL-induced cell injury [34–37]. Furthermore, Adachi et al. showed that S-glutathionylation of sarco/endoplasmic reticulum calcium (Ca^2+^) ATPase (SERCA) induces NO-dependent relaxation that is impaired following irreversible oxidation of key thiol(s) during atherosclerosis, so preventing the activation of SERCA [38]. The same team described the mechanisms of redox-dependent p21ras activation induced by oxLDL in endothelial cells [39]. Actually, protein-S-glutathionylation/deglutathionylation in monocytes and macrophages is thought to be an important signalling mechanism that modulates cell response to oxidative stress in key events of plaque initiation (monocyte recruitment and differentiation) and progression (macrophage activation and death) [40]. Increased serum protein glutathionylation has been also correlated with peripheral atherosclerosis [41]. Furthermore, serum levels of glutathione, homocysteine, and cysteine have been independently associated with cardiovascular risk scores at a population level [42].

Elevated plasma levels of homocysteine are a known risk factor for cardiovascular disease and atherothrombosis [43]. Some of its effects include impaired relaxation of blood vessels mediated by the endothelium-derived NO, thiolation of plasma or endothelial proteins, low-density lipoprotein S-homocysteinylation [44, 45], activation of inflammatory pathways and apoptosis, vascular SMC activation and proliferation, and macrophage activation and differentiation [46–50]. Besides GSH, also homocysteine is involved in extracellular matrix remodelling through activation of latent metalloproteinases [26, 28, 29].

Attempting to investigate the relationship between oxidative stress and plaque progression, we studied sulfhydryl group oxidative modifications of extractable proteins from advanced human atherosclerotic plaques, by means of a differential proteomic approach [51]. The study provided evidence that in unstable carotid plaques, and to a lesser extent in stable ones, there is a prooxidant microenvironment conducive to the formation of ROS- and RNS-mediated protein thiol oxidation products. The sulfhydryl group oxidation observed regarded both filtered (e.g., albumin and transferrin) and topically expressed (e.g., *α*-actin) proteins. Those findings were also corroborated by capillary electrophoresis analysis of LMW thiols bound to extractable proteins that showed higher levels of protein thiolation in unstable plaque extracts. Interestingly, such an increase in protein-bound thiol content was not associated with a concurrent increase in total LMW thiol content. Furthermore, the levels of plasma LMW thiols did not discriminate between patients with stable and unstable plaques, suggesting that the observed differences resulted from oxidative events which take place inside the atherosclerotic plaque [51].

## 4. Human Serum Albumin as a Carrier of Harmful LMW Thiols inside the Atherosclerotic Plaque

Human serum albumin (HSA) is the most abundant plasma multifunctional protein and the major antioxidant in plasma with a concentration (0.8 mM) higher than the other antioxidants by an exponential factor [52]. Its plasma levels have been strongly inversely correlated with both incident coronary heart disease [53, 54] and some carotid plaque oxidation markers (i.e., TBARS and AOPP) detected in advanced lesions [55]. Recently, its redox state has been associated with some indices of atherosclerosis [56]. Furthermore, a positive correlation between serum total homocysteine and HSA-bound homocysteine in hyperlipidaemic patients has been reported [57]. Interestingly, S-thiolation of HSA occurred not only at Cys^34^ but also at other cysteine residues, such as Cys^90^ and Cys^101^ [57].

Albumin accounts for ROS/RNS scavenging, metal ion binding [58], and transport functions for fatty acids [59, 60], nitric oxide, hemin, and drugs [61–63]. It displays also pseudoenzymatic hydrolytic activity of several endogenous and exogenous compounds [64]. Most of the abovementioned functions are due to its unique redox active free cysteine residue (Cys^34^) [65]. Thiol concentration in plasma are lower than in the cells, mostly represented by albumin Cys^34^ residue, which accounts for 80% (500 *μ*mol/L) of total plasma thiols. Due to its reactivity (unexpectedly low Cys^34^ pKa) and its relatively high concentration, it is the preferential target for oxidants and electrophiles. In fact, in blood as well as in extravascular fluids, albumin is susceptible to different oxidative modifications, especially thiol oxidation and carbonylation [66, 67]. Although albumin circulates primarily in its reduced form, about 30–40% of its reactive Cys^34^ residues could be variably oxidized, either reversibly as mixed disulphide with low-molecular-weight thiols [68], S-nitroso Cys [69], or sulfenic acid [70] or irreversibly as sulfinic or sulfonic acid [52] (Figure 1). Furthermore, it has been described that albumin, through its nucleophilic Cys^34^ residue, acts as scavenger for proatherogenic species such as 4-hydroxy-trans-2-nonenal [71].

S-Thiolation of circulating albumin by LMW thiols is the most prevalent Cys^34^ oxidative modification. Although the proinflammatory mechanisms mediated by LMW thiols are not yet completely understood, it was suggested that albumin could act as homocysteine carrier inside the cells where it could exert its noxious effects by altering the redox potential or modifying intracellular proteins resulting in cellular dysfunction [72].

Starting from this interesting assumption, we have hypothesized that circulating albumin may filter into the atherosclerotic plaque where it may release harmful LMW thiols. Some previous results obtained on advanced human carotid plaques extracts showed that about 70% of extractable proteins were of plasma origin, being albumin the most represented [17], and intraplaque LMW thiol content and distribution deeply differed from plasma [73]. In particular, the high intraplaque glutathione levels, probably consequent to red blood cell lysis, a frequently observed event in atherosclerotic lesions, could contribute to plaque fate by perturbing the physiological LMW thiol redox state.

To assess a possible role for albumin as a carrier of LMW thiols inside the plaque, we set up a very sensitive method for the thiolation analysis of both circulating and intraplaque albumin [74]. The method, which allows for the separation and quantitation of all five LMW thiols starting from 3 *μ*g of albumin, consisted of (1) a preanalytical albumin purification from both plasma and plaque extracts by nonreducing SDS-PAGE, (2) in-gel extraction of LMW thiols, and (3) capillary electrophoresis laser induced fluorescence analysis (CE-LIF).

**Figure 2:**
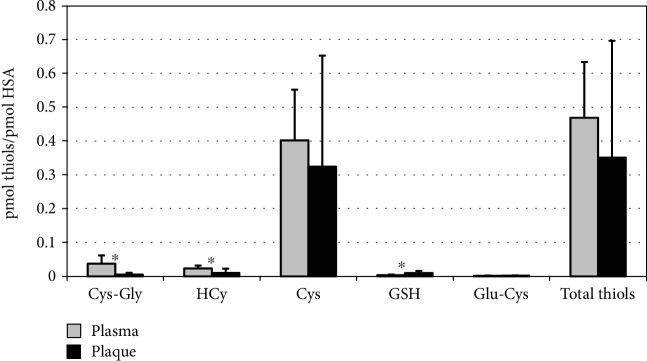
Levels of LMW thiols extracted from both circulating and plaque-filtered HSA, expressed as pmoles/pmoles of albumin, obtained by CE-LIF analysis (from [75]). ^∗^Significant differences between the two HSA forms (*P* < 0.001). Cys-Gly: cysteine-glycine; HCy: homocysteine; Cys: cysteine; GSH: glutathione; Glu-Cys: glutamylcysteine.

This method was applied to the analysis of HSA Cys^34^ thiolation/oxidation on twenty-seven atherosclerotic plaque specimens and the corresponding plasma samples collected from patients undergoing carotid endarterectomy [75]. By this way, we evidenced distinct patterns of thiolation for the two HSA forms with a significant reduction of Cys-Gly (∼7-fold) and HCy (∼2-fold), as well as an increase of GSH (∼2.8-fold) in intraplaque HSA (Figure 2). Overall, following infiltration, albumin releases 16.2 ± 11.2 nmol HCy/g proteins and 32.8 ± 23.9 nmol Cys-Gly/g proteins, which represent the bulk of free HCy and Cys-Gly inside the plaque environment [44]. In this regard, carotid plaques are characterized by very high intraplaque GSH levels, with respect to plasma (225 ± 177 nmol/g proteins vs. 59 ± 15 nmol/g proteins, 23.9% vs. 1.72%), probably due to cell lysis during apoptotic and/or necrotic events, responsible for the different equilibrium among protein-bound LMW thiols and the higher Cys^34^ glutathionylation of the intraplaque form.

These *in situ* evidences have been further supported by recent *in vitro* data [76] showing equilibria among protein-bound LMW thiols and free LMW thiols released in a thiol-free medium. In particular, the Authors, by using commercial HSA with or without previous incubation with HCy and HCy thiolactone, have evidenced that (1) albumin spontaneously releases HCy in a thiol-free medium (and likely in a thiol-poor medium such as the plaque environment) and such event (2) is positively correlated with the levels of albumin homocysteinylation and (3) could be further increased by the presence in the medium of other LMW thiols, such as Cys and GSH.

## 5. Conclusions

Understanding the mechanisms that regulate either plaque stabilization or its evolution towards complication and rupture is essential to prevent the clinical events related to acute artery atherothrombosis.

Within the arterial wall, oxidative modifications could initiate and/or contribute to atherogenesis and plaque development. Among them, the formation of mixed disulfides between protein sulfhydryls and LMW thiols may represent an antioxidant defence mechanism, and it has been suggested as a possible redox regulation mechanism of protein function and cell signalling.

Although several *in vitro* and *in vivo* evidences show a link between protein thiolation and cardiovascular diseases, only scanty data on these oxidative modifications *in situ* (inside atherosclerotic plaque) have been reported so far, leaving the issue at a speculative perspective.

In these respect, in the last years, a step forward has been made by reporting the different sulfhydryl oxidation/thiolation of plaque proteins in relation to stable/unstable advanced atherosclerotic lesions and demonstrating that serum albumin, the main plasma protein filtered in plaque, represents a carrier of LMW thiols inside the atherosclerotic lesions, where it releases harmful quantities of homocysteine, probably contributing to plaque progression. However, some aspects, including the equilibria between LMW thiols and protein sulfhydryls, and the effects of specific protein thiolation on their functions remain to be elucidated. They could provide further insight into the relevance of oxidative modifications in atherosclerotic plaque development and progression towards advanced lesions and, surely, deserve further studies.
